# Constitutive and Regulated Shedding of Soluble FGF Receptors Releases Biologically Active Inhibitors of FGF-2

**DOI:** 10.3390/ijms22052712

**Published:** 2021-03-08

**Authors:** Anne Hanneken, Maluz Mercado, Pamela Maher

**Affiliations:** Department of Molecular Medicine, The Scripps Research Institute, 10550 North Torrey Pines Road, La Jolla, CA 92037, USA; maluzm@hotmail.com (M.M.); pmaher@salk.edu (P.M.)

**Keywords:** ectodomain shedding, FGF, soluble receptors, downregulation

## Abstract

The identification of soluble fibroblast growth factor (FGF) receptors in blood and the extracellular matrix has led to the prediction that these proteins modulate the diverse biological activities of the FGF family of ligands in vivo. A recent structural characterization of the soluble FGF receptors revealed that they are primarily generated by proteolytic cleavage of the FGFR-1 ectodomain. Efforts to examine their biological properties are now focused on understanding the functional consequences of FGFR-1 ectodomain shedding and how the shedding event is regulated. We have purified an FGFR-1 ectodomain that is constitutively cleaved from the full-length FGFR-1(IIIc) receptor and released into conditioned media. This shed receptor binds FGF-2; inhibits FGF-2-induced cellular proliferation; and competes with high affinity, cell surface FGF receptors for ligand binding. FGFR-1 ectodomain shedding downregulates the number of high affinity receptors from the cell surface. The shedding mechanism is regulated by ligand binding and by activators of PKC, and the two signaling pathways appear to be independent of each other. Deletions and substitutions at the proposed cleavage site of FGFR-1 do not prevent ectodomain shedding. Broad spectrum inhibitors of matrix metalloproteases decrease FGFR-1 ectodomain shedding, suggesting that the enzyme responsible for constitutive, ligand-activated, and protein kinase C-activated shedding is a matrix metalloprotease. In summary, shedding of the FGFR-1 ectodomain is a highly regulated event, sharing many features with a common system that governs the release of diverse membrane proteins from the cell surface. Most importantly, the FGFR ectodomains are biologically active after shedding and are capable of functioning as inhibitors of FGF-2.

## 1. Introduction

Mounting evidence indicates that ectodomain shedding of cell surface proteins is an essential element of normal cellular behavior at multiple stages of growth and differentiation (for reviews, see [1,2,3,4,5]). Among its many roles, ectodomain shedding releases mature growth factors and coreceptors that regulate cellular proliferation and differentiation [6,7], induces conformational changes that lead to receptor activation [8], and alters the display of cell adhesion molecules which promote or inhibit cell–cell adhesion and migration [9,10,11]. Interference with the normal process of ectodomain shedding in *Drosophila* and mammals leads to a wide variety of developmental and functional defects, emphasizing the importance of shedding throughout the phylogenetic tree [6,8,10,12]. 

The shedding of cell surface receptors is a rapid method for regulating the biological activities of growth factors. Shedding leads to receptor downregulation, decreasing the number of full-length receptors on the cell surface that can respond to ligand binding. At the same time, the shed receptor ectodomains retain an affinity for their ligands, which allows them to function as competitive inhibitors in the pericellular environment. Together, these complementary processes inhibit cytokine activity in physiologically significant ways. The biological relevance of this mechanism is shown by the finding that germline mutations in the 55 kDa tumor necrosis factor (TNF) receptor cause a variety of inherited autoimmune inflammatory syndromes that are thought to be the result of both reduced levels of the circulating, soluble TNF receptors and impaired TNF receptor clearance [13]. Furthermore, medical treatments with recombinant soluble receptors can block the biological activity of cytokines in vivo, as shown by the reduction of retinal edema in patients treated with a soluble chimeric vascular endothelial cell growth factor (VEGF) receptor (aflibercept, Eylea^TM^) [14] and the reduction of arthritic joint damage in patients treated with a dimeric, soluble TNF receptor immunoadhesin (Etanercept, Enbrel^TM^) [14,15,16].

Studies with mutant Chinese hamster ovary (CHO) cells have shown that the mechanisms regulating the shedding of diverse cell surface proteins have many features in common [17]. Both constitutive and activated cleavage pathways have been identified, with the latter being activated by cytokines, growth factors, activators of protein kinase C (PKC), and calcium ionophores [18,19,20]. A variety of enzymes, which are members of the metzincin family of zinc-dependent proteinases, are responsible for ectodomain cleavage. These include the MMPs (matrix metalloproteases) and the ADAM (a disintegrin and metalloprotease) family members, ADAM-17 (tumor necrosis factor converting enzyme; TACE), ADAM-12, ADAM-9, and ADAM-10, among others [8,21,22,23,24]. The constitutive and activated cleavage pathways are independent of a specific consensus sequence and are regulated via a variety of different protein kinase pathways [19,25]. 

We have proposed that ectodomain shedding of the high affinity fibroblast growth factor (FGF) receptors (FGFR-1-4) is an essential mechanism for controlling the diverse biological activities of the FGF family of growth factors during growth and development [26,27,28]. The FGFs are a family of highly conserved and tightly regulated pleiotropic growth factors which function in diverse biological processes to induce cellular differentiation and proliferation, promote mesodermal induction and patterning, regulate limb outgrowth and multi-organ development, and enhance neovascularization and wound repair [29,30,31,32,33,34]. The prototype member of the FGF family is FGF-2, which is widely distributed in cells and the extracellular matrix of both proliferating and nonproliferating cells [35]. The ubiquitous distribution of the FGFs, especially FGF-2, surrounding cells that respond to FGF-2 in vitro, but are quiescent in vivo, emphasizes that multiple levels of regulation exist, which control the biological activity of the FGFs. Mechanisms that have been proposed to explain this phenomenon include the enzymatic release of FGF-2 from receptors in the extracellular matrix [36], post-translational modifications of the FGFs [37], alternative signaling pathways [38], specific temporal and spatial FGF receptor expression patterns [39], and the shedding of both low and high affinity FGF receptors [7,25,27,39,40]. 

The FGF receptors consist of two classes of cell surface moieties—low affinity heparan sulfate proteoglycans and a gene family of four high affinity tyrosine kinase receptors (FGFR-1-4)—which encode multiple isoforms that are generated by the alternative splicing of mRNA transcripts [40,41,42]. The major class of FGF binding, transmembrane heparan sulfate proteoglycans, known as the syndecans, are constitutively shed from cell membranes and are capable of modulating the biological activity of FGF-2 through interactions with the high affinity FGF receptors [43]. The syndecans are shed in response to thrombin and growth factors, such as epidermal growth factor (EGF), and are present in wound fluids [44,45]. The biological activity of the syndecans is regulated by heparinase-like proteases, which convert the proteoglycans from inhibitors to activators of FGF-2 [7].

**Figure 1:**
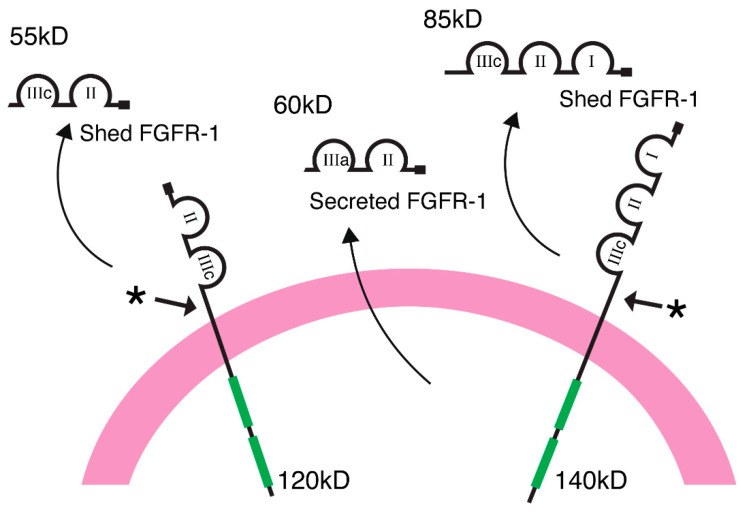
Schematic illustration of the full-length transmembrane FGFR-1 receptors showing the two and three Ig-like extracellular domains (IIIc) that are proteolytically cleaved from the cell surface. A two-loop ectodomain of FGFR-1 is expressed from an alternatively spliced transcript of mRNA and secreted into the extracellular milieu without proteolytic processing.

We have identified a class of high affinity, soluble FGF receptors in blood and other biological fluids, and in the extracellular matrix of vascular endothelial cells [27,28,35,46]. This group of soluble receptors consists of at least five isoforms of the high affinity FGF receptor gene family, including a two and three Ig-like loop ectodomain of FGFR-1(IIIb) and FGFR-1(IIIc), and a unique two Ig-domain secreted isoform called FGFR-1(IIIa) (Figure 1). Analysis of the carboxyl-terminus indicates that the two and three Ig-like loop ectodomains of FGFR-1 are generated by proteolytic cleavage of the full-length cell-surface FGF receptors, eight amino acids proximal to the transmembrane domain [26,47]. 

The biological activities of the native, circulating soluble FGF receptors have not been reported. However, recombinant isoforms of the extracellular domains of FGFR-1 and FGFR2 inhibit FGF-2-induced proliferation [48] and block FGFR2 signaling [34], supporting the hypothesis that soluble FGF receptors are modulators of FGFs in vivo. In order to study the properties of the soluble receptors further, we turned to a cell-based system to obtain substantial quantities of the proteolytically cleaved ectodomain that is derived from the extracellular domain of the full-length cell surface receptor and the secreted receptor that is produced by the expression of an alternatively spliced mRNA transcript.

In this paper, we focus on three important questions: (i) Is the FGFR-1 ectodomain biologically active after shedding? (ii) Is the shedding event regulated in vitro? (iii) Does the shedding process share similarities with the common system responsible for the shedding of other transmembrane proteins from the cell surface? Here, we show that the proteolytically cleaved three Ig-like loop form of the FGFR-1 ectodomain inhibits the activity of FGF-2 by competing with high affinity FGF receptors for ligand binding and blocks FGF-2-induced cell proliferation and in-vitro angiogenesis. Shedding of the FGFR-1 ectodomain is regulated by both ligand binding and activators of PKC and the two signaling pathways appear to be independent of each other. Full-length FGFR-1 receptors decrease as the shedding of the FGFR-1 ectodomain increases, consistent with receptor downregulation. Constitutive, ligand-activated, and PKC-activated shedding appears to be regulated by a metalloprotease family member. FGF receptor mutants, with deletions and substitutions intended to disrupt the conformational stability at the putative cleavage site, are still shed in proportion to the expression levels of the high affinity receptor. Therefore, the release of a biologically active FGFR-1 ectodomain is both a constitutive and tightly regulated event, sharing many features with the system that regulates the release of other diverse membrane proteins from the cell surface. 

## 2. Results

### 2.1. Identification of a Shed FGFR-1 Ectodomain in the Conditioned Media of Transfected COS 7 Cells

**Figure 2:**
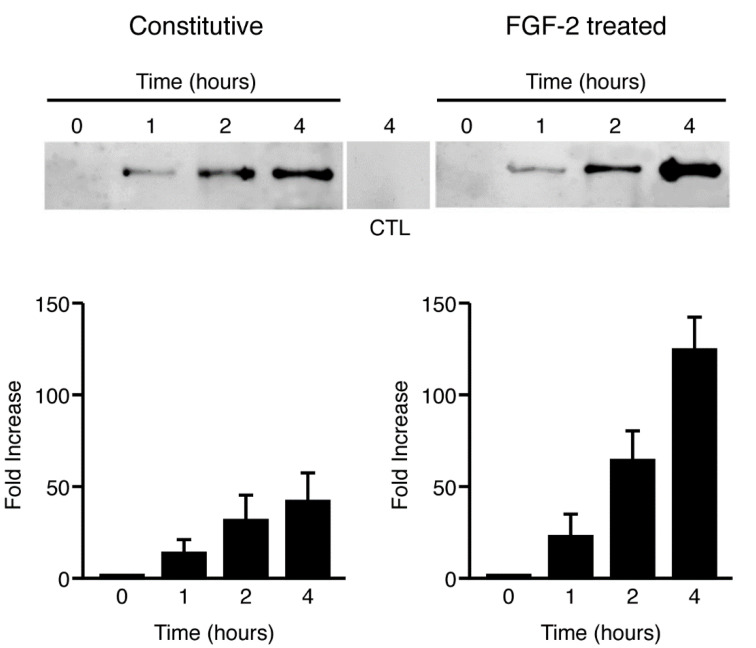
Constitutive and ligand-induced FGFR-1 ectodomain shedding. COS 7 cells transfected with full-length FGFR-1 cDNA were placed in serum-free media for up to four hours, in the presence or absence of recombinant, human fibroblast growth factor (FGF)-2. After collecting the conditioned media, the FGFR-1 ectodomain was precipitated with WGA-Sepharose, eluted with sample buffer, and analyzed by SDS-PAGE and immunoblotting with an antibody to the extracellular domain of FGFR-1 (Mab6). The left panel shows the time course of the constitutive release of an 85 kDa FGFR-1 ectodomain and the right lane shows the time course of the activated release of the FGFR-1 ectodomain in the presence of FGF-2. The negative control consists of the 4 h time point of media from untransfected cells. The densities of the bands were measured with NIH Image software and the percent change in intensity of the bands was calculated using the control lane as a reference for comparison. Standard deviations were calculated for each individual band by the imaging software.

To explore the possibility that the FGFR-1 ectodomain is shed constitutively from cells that express full-length FGFR-1, we utilized a transient transfection assay, which has previously been used to evaluate the release of a variety of ectodomains from cell surface proteins [17]. We analyzed the serum-free conditioned media from COS 7 cells that were transfected with the full-length, three Ig-like domain isoform of FGFR-1 (see Figure 2). Using a specific antibody (Mab6) raised against the extracellular domain of FGFR-1, we identified a shed FGFR-1 ectodomain in the conditioned media of the FGFR-1-transfected COS 7 cells, but not in the media of untransfected or vector transfected COS 7 cells. The FGFR-1 ectodomain was shed into the conditioned media constitutively, increasing in a time-dependent manner (Figure 2, left). The molecular weight of the shed receptor was between 70 and 85 kDa, which is identical to the size of the native, three Ig-like domain form of soluble FGFR-1 that has been purified from blood [28].

Growth factors and cytokines have been shown to induce ectodomain shedding by the activation of specific signaling cascades [19]. To examine whether FGF-2 could enhance shedding of the FGFR-1 ectodomain in an autocrine manner by binding to the cell surface FGF receptors and activating downstream signaling pathways, we added exogenous FGF-2 (0.5 ng/mL) to the serum-free media and repeated the time course. We found that shedding of the FGFR-1 ectodomain was increased by the addition of FGF-2, most notably after four hours (Figure 2, right). Image analysis of the four-hour time points revealed that the increase in shedding in the presence of FGF-2 was approximately three-fold over the level of constitutive shedding. 

### 2.2. Ligand-Induced Shedding Leads to a Decrease in Full-Length FGFR-1 Receptors

**Figure 3:**
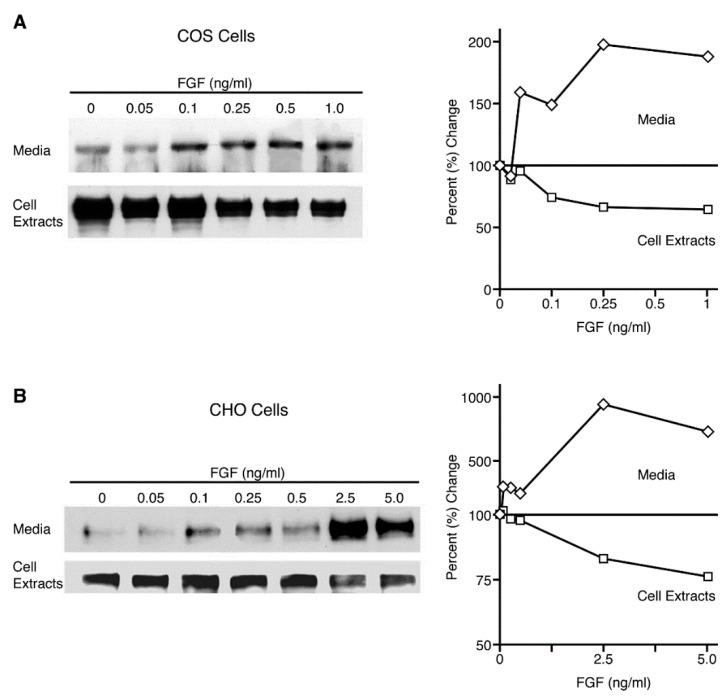
Ligand-induced ectodomain shedding downregulates full-length receptors in cell extracts. (**A**) COS 7 cells, transiently transfected with full-length FGFR-1(IIIc) cDNA, and (**B**) CHO cells, stably transfected with full-length FGFR-1(IIIc) cDNA, were placed in serum-free media in the presence of different concentrations of FGF-2. Conditioned media and cell extracts were collected after two hours. The shed FGFR-1 ectodomain was precipitated with WGA-Sepharose. Full-length FGFR-1 in the cell lysates was precipitated with an antibody to the C-terminus of FGFR-1 and/or extracted with sample buffer. The samples were analyzed by SDS-PAGE and immunoblotting with Mab6. The upper and lower panels show the release of the FGFR-1 ectodomain from COS 7 and CHO cells, respectively, and the corresponding decrease in the level of the full-length receptor. The densities of the bands were measured with NIH Image software and the percent change in intensity of the bands was calculated using the control lane (0 ng/mL) as a reference for comparison.

We explored the dose dependency of ligand-induced shedding in COS 7 and CHO cells to determine whether this event was cell-type specific or representative of a more general phenomenon. Using transiently transfected COS 7 cells and the stably transfected CHO cell line FGFR-1/pcDNA3, we observed an increase in the shedding of the FGFR-1 ectodomain in both cell types after stimulation with FGF-2 (Figure 3A,B, upper). Ligand-induced ectodomain shedding was dose-dependent, reaching a maximum at slightly different concentrations of FGF-2 in the two cell types. 

Since the shedding of cell surface proteins has been proposed to be a mechanism of receptor downregulation [3], we examined the cell lysates to determine whether the full-length cell surface FGF receptors decreased as the shedding of the FGFR-1 ectodomain increased. We found that FGF-induced shedding of the FGFR-1 ectodomain led to a dose-dependent decrease in the 140 kDa full-length cell surface FGF receptor in both cell lysates (Figure 3A,B, lower). These results support the concept that shedding of the FGFR-1 ectodomain could play a role in the downregulation of cell surface FGF receptors after ligand binding.

### 2.3. Purification of the Soluble FGFR-1 Ectodomains

**Figure 4:**
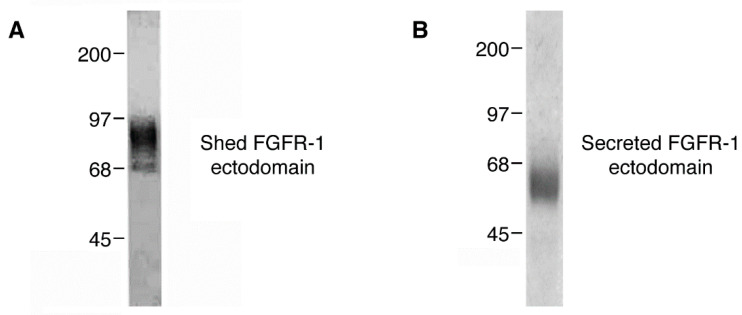
Purification of the soluble FGFR-1 ectodomains. (**A**) Coomassie staining of the shed FGFR-1 ectodomain purified from CHO cell conditioned media. (**B**) Coomassie staining of the secreted FGFR-1 ectodomain purified from CHO cell conditioned media.

With the evidence that FGFR-1 ectodomain shedding is a regulated event, we focused on the question of whether the soluble FGFR-1 ectodomains are biologically active. We passed FGFR-1 CHO cell conditioned media over an FGF-2/heparin-Sepharose affinity column to determine whether the soluble FGFR-1 ectodomains could bind FGF-2. Both the shed and secreted FGFR-1 ectodomains bound avidly to the FGF-2/heparin-Sepharose column. Coomassie blue staining of the eluants revealed molecular weights of 70–85 kDa and 55–60 kD for the shed FGFR-1 and the secreted FGFR ectodomains, respectively (Figure 4A,B). The purified FGFR-1 ectodomains had the same electrophoretic characteristics as the native, 85 kDa and 55 kD soluble FGF receptors isolated and purified from blood [26,28]. 

### 2.4. The Shed FGFR-1 Ectodomain Functions as a Biologically Active Inhibitor of FGF-2

**Figure 5:**
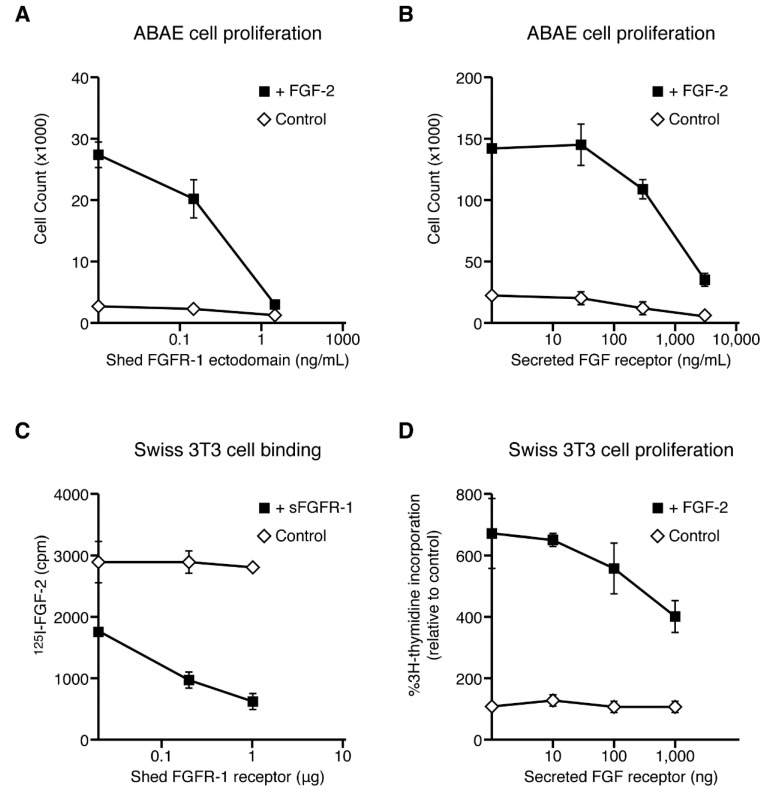
The soluble FGFR-1 receptors inhibit the biological activity of FGF-2. (**A**,**B**) Adult aortic endothelial cell proliferation is inhibited by soluble FGF receptors. Cells were plated in 24-well chambers in complete medium with 0.125 ng/mL FGF in the absence or presence of increasing concentrations of the shed (**A**) or secreted (**B**) FGFR-1 ectodomain on days 1 and 3. After five days in culture, the cells were trypsinized and counted with a Coulter Counter. (**C**) Soluble FGF receptors inhibit FGF-2 binding to cell surface FGF receptors in the ^125^I-FGF-2 ligand binding assay. Swiss 3T3 cells were grown to confluence in 24-well plates. The cells were labeled for 2 h at 4 °C with ^125^I-FGF-2 (2 ng/mL) in the absence or presence of increasing concentrations of the shed FGFR-1 ectodomain. High affinity FGF-2 binding was determined after removing FGF-2 bound to low affinity sites with a high salt wash. Results are presented as the decrease in ^125^I-FGF-2 binding to 3T3 cell surface FGFR-1 receptors in the presence of the shed ectodomain. (**D**) Swiss 3T3 cell ^3^H-thymidine incorporation assays. Swiss 3T3 fibroblasts were grown to confluence in 96-well dishes and switched to low serum media for 2 days in DME (Dulbecco’s Modified Eagle’s Media) with 0.5% calf serum. The rate of DNA synthesis was measured 24 h after the addition of FGF-2 and/or secreted FGFR-1 and 5 h after incubation with 0.2 mCi/well of [methyl-^3^H]thymidine (6.7 Ci/mole, ICN). The cultures were then processed for scintillation counting, as previously described [49].

We tested the inhibition of FGF-2 activity with two forms of soluble FGFR-1 receptors: A proteolytically shed form of soluble FGFR-1 and a secreted soluble FGFR, expressed from an alternatively spliced transcript that lacks a transmembrane domain [26]. We used a classic FGF-2-induced aortic endothelial cell proliferation assay to test the effects of soluble FGF receptors on FGF-2-induced proliferation, and an ^125^I-FGF-2 receptor-binding assay to address the mechanism of this activity. We found that both the shed and secreted FGFR-1 ectodomains inhibited FGF-2-induced aortic endothelial cell proliferation in a dose-dependent manner (Figure 5A,B). 

To explore the mechanism behind this inhibition, we switched to Swiss 3T3 cells due to the higher number of cell surface FGF receptors in this cell line. We measured ^125^I-FGF-2 binding to high affinity, cell surface receptors in both the presence and absence of the shed FGFR-1 ectodomain. We found that the shed FGFR-1 ectodomain effectively reduced ^125^I-FGF-2 binding to high affinity FGF receptors (Figure 5C, left), supporting the interpretation that the mechanism of inhibition is due to competition between soluble and cell surface FGF receptors for FGF-2 binding. To further support this interpretation, we examined the effect of the secreted FGFR-1 ectodomain on cellular proliferation in Swiss 3T3 cells, using a ^3^H-thymidine incorporation assay. Similar to the effects of soluble FGFR-1 receptors in the ABAE cell proliferation assays, the secreted FGFR-1 receptor inhibited FGF-2-induced 3T3 cell proliferation to a similar degree as the inhibition of ^125^I-FGF-2 binding (Figure 5D, right). These results support the interpretation that the shed and secreted FGFR-1 ectodomains inhibit the biological activity of FGF-2 in multiple cell types and compete with the high affinity, cell surface FGF receptors for binding to FGF-2.

### 2.5. The Secreted FGFR-1 Receptor Inhibits Capillary Tube Formation in Collagen Gels

**Figure 6:**
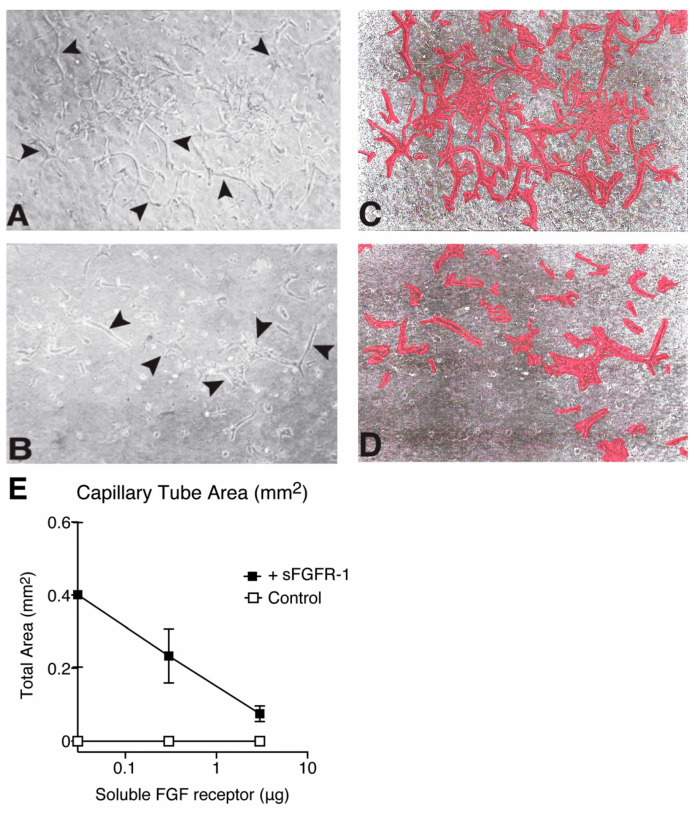
Inhibition of in-vitro angiogenesis and capillary tube formation by soluble FGFR-1 receptors. Phase-contrast images of ABAE cells forming three-dimensional capillary structures (arrows) in collagen gels grown with FGF-2 (500 pg) in the absence (**A**) or presence (**B**) of the secreted FGFR-1 receptor (300 ng). Bar = 200 um. Panels (**C**,**D**) show the corresponding capillary tube structures highlighted in red. (**E**) The area of capillary tube formation was quantified using ImageJ.

Using a well-known model of in vitro angiogenesis, we tested the ability of the secreted FGF receptor to block FGF-2-induced capillary tube growth in collagen gels [50]. Within seven days of growth, the ABAE cells cultured in the presence of FGF-2 invaded the collagen gel and formed three-dimensional capillary tubes with branching patterns. Cells cultured in the absence of FGF-2 showed no evidence of capillary tube formation. Cells cultured with FGF-2 and increasing concentrations of the secreted FGFR-1 ectodomain showed progressively less capillary tube formation (Figure 6A–E), demonstrating that the soluble FGF receptors inhibit FGF-2-induced angiogenesis in the extracellular matrix in a dose-dependent manner. 

### 2.6. Inhibition of FGFR-1 Ectodomain Shedding by Metalloprotease Inhibitors

**Figure 7:**
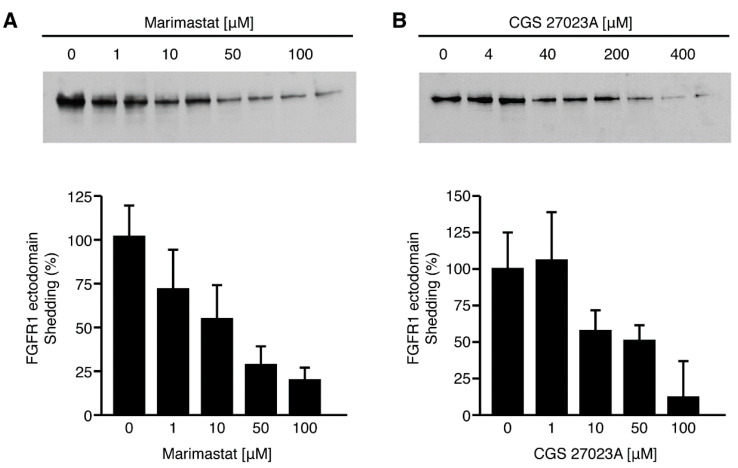
Inhibition of constitutive FGFR-1 ectodomain shedding by MMP inhibitors. COS 7 cells transiently transfected with FGFR-1(IIIc) were placed in serum-free media in the presence of different concentrations of (**A**) marimastat and (**B**) CGS 27023A, as indicated. The samples were run in duplicate. The conditioned media was collected after an overnight incubation and the shed FGFR-1 ectodomain was precipitated with WGA-Sepharose. The samples were analyzed by SDS-PAGE and immunoblotting with Mab6. The densities of the bands were measured with NIH Image software and the percent change in intensity of the bands was calculated using the control lane (0 uM) as a reference for comparison. Standard deviations were calculated for each individual band by the imaging software.

A diverse group of membrane proteins are shed by a mechanism which is sensitive to metalloprotease inhibitors [17]. To examine whether the constitutive and ligand-activated FGFR-1 ectodomain shedding is also dependent on this system, we tested the effect of metalloprotease inhibitors on the release of the FGFR-1 ectodomain in COS 7 cells. Cells treated with the metalloprotease inhibitors—marimastat and CGS27023A—showed a dose-dependent decrease in constitutive FGFR-1 ectodomain shedding (Figure 7). FGF-2-activated shedding was also inhibited. There was no evidence of cytotoxicity (data not shown).

### 2.7. FGFR-1 Ectodomain Shedding Is Not Inhibited by Mutations Surrounding the Cleavage Site

**Figure 8:**
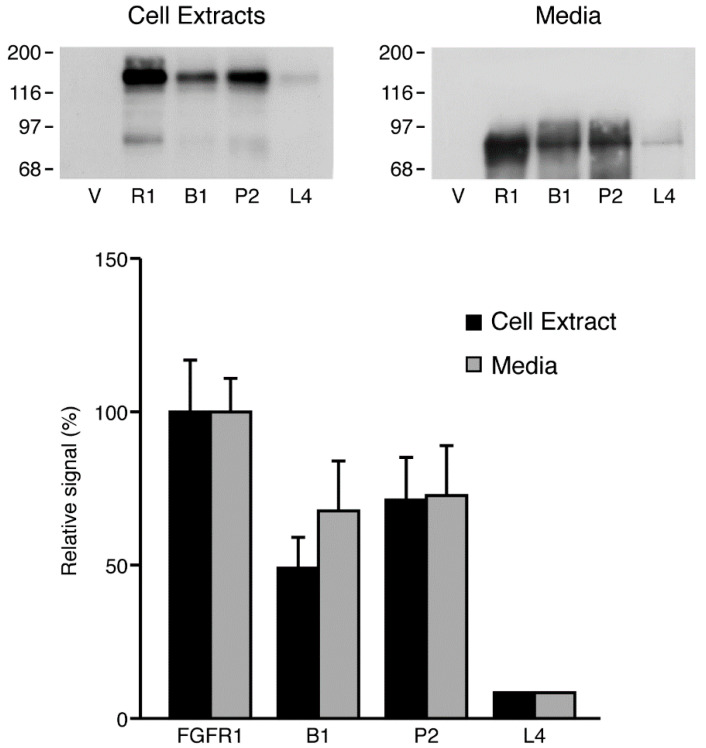
The effect of FGFR-1 mutations on ectodomain shedding. COS 7 cells were transfected with vector alone (pcDNA3) or FGFR-1 constructs, as indicated, including FGFR-1 (wild-type), FGFR-1/B1, FGFR-1/P2, and FGFR-1/L4. The conditioned media and cell extracts were collected after an overnight incubation. The shed FGFR-1 ectodomains were precipitated with WGA-Sepharose and analyzed by SDS-PAGE and immunoblotting with Mab6. The densities of the bands were evaluated using NIH Image software.

The putative cleavage site of the FGFR-1 ectodomain is located between Val-Met, eight amino acids from the transmembrane domain [26,47]. We constructed an FGFR-1 mutant containing a deletion at the putative cleavage site and analyzed the degree of FGFR-1 ectodomain shedding following the transfection of COS 7 cells with the mutant FGFR-1 construct. We found that a seven amino acid deletion surrounding the putative cleavage site did not prevent shedding of the FGFR-1 ectodomain (Figure 8). Indeed, the relative degree of ectodomain shedding was not significantly different from the wild type receptor, adjusting for the 50% lower level of expression of the full-length receptor mutant (see the histogram in Figure 8). We constructed and examined two additional mutants:FGFR-1/P2, which contains a 10 amino acid deletion with a proline-glycine substitution and FGFR-1/L4, which contains a 14 amino acid deletion in the region of the putative cleavage site. Both of these mutants were also cleaved. Although the extent of ectodomain shedding was lower than with the wild type receptor, this difference was eliminated when the level of shed receptor was compared to the overall level of expression of the full-length, mutant receptors. These findings indicate that FGFR-1 ectodomain shedding is not inhibited by mutations containing deletions and substitutions at the putative cleavage site, which is a feature that is relatively common for other cell surface proteins.

### 2.8. Constitutive FGFR-1 Ectodomain Shedding Is Activated by TPA

**Figure 9:**
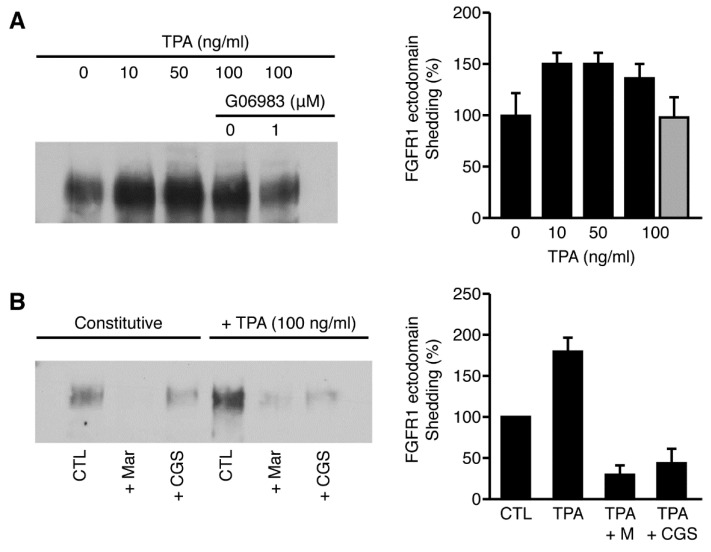
12-O-tetradecanoyl phorbol 13-acetate (TPA) enhances FGFR-1 ectodomain shedding. (**A**) COS 7 cells transiently transfected with FGFR-1(IIIc) were placed in serum-free media in the presence of different concentrations of TPA plus or minus G_0_6983. The conditioned media was collected after a 36 h incubation and the shed FGFR-1 ectodomain was precipitated with WGA-Sepharose. The samples were analyzed by SDS-PAGE and immunoblotting with Mab6. The densities of the bands were measured with NIH Image software and the percent change in intensity of the bands was calculated using the control lane (0 ng/mL) as a reference for comparison. Standard deviations were calculated for each individual band by the imaging software. (**B**) COS 7 cells transiently transfected with FGFR-1(IIIc) were placed in serum-free media in the presence or absence of 100 ng/mL TPA and the metalloprotease inhibitors marimastat (25 mM) and CGS 27034A (50 mM). The conditioned media was collected after a 36 h incubation and the FGFR-1 ectodomain was analyzed as described above.

Several studies have demonstrated that the constitutive shedding of diverse transmembrane proteins is activated by protein kinase C signaling pathways [17]. To determine whether activators of protein kinase C enhance shedding of the FGFR-1 ectodomain, we examined shedding of the FGFR-1 ectodomain in the presence of the PKC activator—TPA. FGFR-1-transfected COS 7 cells were treated with TPA in the presence or absence of G_0_6983, which is a specific inhibitor of all PKC isoforms except PKCμ [51]. We found that TPA activated FGFR-1 ectodomain shedding at concentrations ranging from 10 to 100 ng/mL and that this enhancement was abolished in the presence of G_0_6983. (Figure 9A). These results indicate that shedding is dependent on cellular signaling mechanisms and the activation of PKC pathways.

To determine whether both constitutive shedding and protein kinase C-induced shedding were sensitive to metalloprotease inhibitors, we examined the effect of marimastat and CGS27023A on constitutive and TPA-activated cleavage. We found that marimastat and CGS27023A abolished constitutive and TPA-activated shedding (Figure 9B), suggesting that both constitutive, ligand-induced and PKC-activated shedding pathways involve a member of the metalloprotease family of enzymes.

### 2.9. FGF-2-Activated Shedding Is Blocked by a Specific Inhibitor of the FGF Receptor Tyrosine Kinase, But Not by a PKC Inhibitor

**Figure 10:**
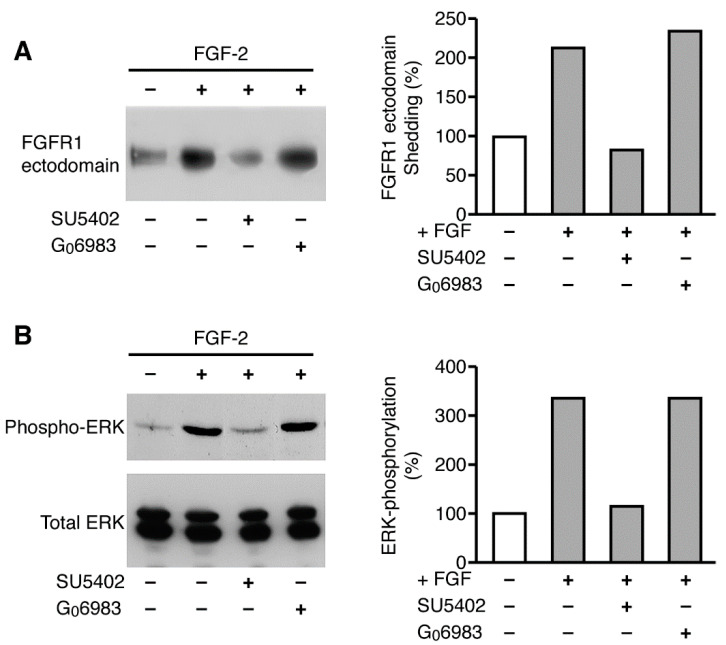
FGF-2-activated shedding is blocked by an FGF receptor tyrosine kinase inhibitor. CHO cells were placed in serum-free media in the presence of FGF-2 and the FGF receptor tyrosine kinase inhibitor SU5402, or the PKC inhibitor G_0_6983. Conditioned media and cell extracts were collected after two hours. The shed FGFR-1 ectodomain was precipitated with WGA-Sepharose. The cells were extracted directly into SDS-PAGE sample buffer. The samples were analyzed by SDS-PAGE and immunoblotting with Mab6 (**A**) or antibodies to phospho-ERK and total ERK (**B**) to test for the inhibition of FGFR-1 activation by SU5402.

We also examined whether stimulation of the FGF receptor tyrosine kinase signaling pathway was necessary for ligand-activated FGFR-1 ectodomain shedding. We analyzed the levels of the shed FGFR-1 ectodomain in the conditioned media of FGFR-1 CHO cells treated with FGF-2 in the presence or absence of a specific inhibitor of the FGF receptor tyrosine kinase—SU5402 [52]. We found that FGF-2 induced the ectodomain shedding of FGFR-1 and that SU5402 reduced FGF-2-activated FGFR-1 ectodomain shedding to the level observed with constitutive shedding (Figure 10A). Controls showed that FGF-2-activated shedding was associated with ERK phosphorylation, which was blocked by the FGFR-1 tyrosine kinase inhibitor SU5402 (Figure 10B). These data suggest that ligand-activated FGFR-1 ectodomain shedding occurs through a mechanism that involves FGF receptor tyrosine kinase signaling (Figure 10B).

Finally, we investigated whether FGF-2-activated FGFR-1 ectodomain shedding was regulated by PKC signaling. We found that the PKC inhibitor G_0_6983 did not reduce FGF-2-activated shedding of the FGFR-1 ectodomain (Figure 10A), although it did block TPA-stimulated shedding (Figure 9A). This result demonstrates that the ligand-activated shedding pathway is independent of the PKC cleavage pathway and that multiple stimuli can converge independently on this target.

## 3. Discussion

In this investigation, we purified a constitutively shed FGFR-1 ectodomain, which is the three Ig-like domain form of FGFR-1(IIIc), to demonstrate that circulating FGFR-1 ectodomains are biologically active inhibitors of FGF-2. Specifically, we have shown that the shed FGFR-1 ectodomain binds to an FGF-2/heparin-Sepharose affinity column, blocks FGF-2-induced proliferation in endothelial cells and 3T3 fibroblasts, inhibits in vitro angiogenesis and capillary tube formation, and competes with high affinity cell surface FGFR-1 receptors for ligand binding. These data demonstrate that the FGFR-1 ectodomain can still bind ligands after proteolytic shedding from cell surface receptors and supports the fundamental hypothesis that the shed FGFR-1 ectodomains in blood may function as circulating inhibitors of the FGFs in vivo. In contrast to previous studies that utilized genetically engineered soluble receptors or ectodomain fusion proteins, this report is the first demonstration that a natural and constitutively shed FGFR-1 ectodomain is biologically active. 

This report also demonstrates that FGFR-1 ectodomain shedding shares many features with the common mechanism that governs the release of other cell surface receptors, cell adhesion molecules, and growth factors. The finding that constitutive and regulated shedding occurs in at least two different cell types is consistent with the idea that FGFR-1 shedding is not restricted to a specific cell type and is representative of a more generalized phenomenon which occurs in multiple types of cells sharing common pathways. Of particular interest is the question of which signaling pathways are involved in these shedding events and whether they are the same or different. Although all the pathways involved in FGFR-1 ectodomain shedding are not known, our data show that at least two independent pathways are involved: A ligand-activated pathway which requires activation of the FGF receptor tyrosine kinase and another pathway involving PKC activation. While the activation of receptor tyrosine kinases has been implicated in the shedding of unrelated transmembrane proteins [19], our findings have added significance because FGFR-1 shedding appears to result from FGF-2 binding to and activation of its own receptor. How activation of the FGF receptor leads to the shedding of its receptor ectodomain is not known at this time. 

In addition, the finding that FGF-2 enhances shedding of the ectodomain of its high affinity receptor is of special interest because it suggests that FGFR-1 ectodomain shedding could be an autocrine feedback mechanism for downregulating FGFR-1 receptors after ligand binding. Therefore, the FGFR-1 receptor joins a group including the TNF receptor and the CSF-1 receptor, which use this mechanism to rapidly modulate their response to ligands in the pericellular environment [3,53,54]. Whether or not the truncated cytoplasmic domain persists after ectodomain shedding and has any role in the cell remains to be investigated.

Our results are consistent with the proposal that a member of the metalloprotease family regulates FGFR-1 ectodomain shedding. The specific enzyme involved in these cleavage pathways is not yet known, but candidates include members of the ADAMs family of metalloproteases, particularly ADAMs-12, -17, -10, and -9, which are responsible for the shedding of multiple cell surface proteins, including tumor necrosis factor-alpha (TNF-α), transforming growth factor (TGF-α), L-selectin, amyloid precursor protein (APP), and heparin-binding epidermal growth factor-like growth factor (HB-EGF) [2,22,23,24,55]. These enzymes are inhibited by hydroxamate compounds, such as marimastat and CGS 27023A, which interfere with zinc binding to the metalloprotease domain [56]. In our study, the concentrations of hydroxamate-based MMP inhibitors which blocked FGFR-1 ectodomain shedding are within the µM range shown to inhibit specific ADAM family members in other ectodomain shedding systems [56]. Studies are currently in progress to determine whether FGFR-1 ectodomain shedding can be induced by ADAM-17 (TACE) or other ADAM family members.

Our group attempted, albeit unsuccessfully, to create an FGFR-1 mutant which was resistant to ectodomain shedding. The introduction of successively larger deletions and substitutions failed to induce structural changes that blocked cleavage without interfering with the normal expression levels of the FGF receptor. Even with the substitution of amino acid sequences from other transmembrane receptors that are not cleaved under normal conditions, such as betaglycan [57], we were unable to generate a noncleavable receptor. We conclude that the factors that promote FGFR-1 shedding supersede the amino acid sequence in the cleavage region. These findings are consistent with the proposal that ectodomain shedding is more dependent on factors that regulate access of the protease to the cleavage site than on the actual consensus sequence itself [58]. 

The primary goal of this study was to focus on the biological activities of the soluble FGF receptors and introduce the mechanisms that regulate shedding. There are some limitations in this study. First, while there is broad cross-reactivity of MMPs across multiple species, there may be some differences in the mechanisms regulating proteolytic cleavage of the human FGF receptors in COS 7 and CHO cells due to species differences. This possibility and other cell-type specificity questions can be investigated in future studies. Second, the shedding mechanisms in this study point to proteases that have similarities with the ADAMs family of metalloproteases, which are key regulators of growth and differentiation. These specific family members deserve additional study as regulators of FGF-2-induced endothelial cell growth and regulation in blood and the extracellular matrix. Third, the downregulation of FGFR-1 and other cell surface receptors can be achieved through multiple mechanisms, from ectodomain shedding to endocytosis-mediated receptor degradation. The relative importance of ectodomain shedding compared to endocytosis-mediated receptor degradation should be examined further.

In summary, this report joins two other studies that support the emerging role of FGF receptor shedding as a regulatory mechanism for the FGF growth factor family in vivo. Transgenic mice that overexpress a secreted FGFR2 ectodomain during development develop severe phenotypic abnormalities that are associated with a disturbance in FGF signaling during patterning and organogenesis [34]. Secondly, shed syndecan proteoglycans, which are low affinity FGF receptors purified from wound fluids in vivo, regulate the mitogenic activity of FGF-2 [7,44,45]. It is tempting to speculate that the shed FGFR-1 ectodomain may interact with the shed syndecan proteoglycans in a synergistic manner to regulate the biological activity of FGF-2 in vivo. Future studies are needed to examine this in more detail. Taken together, these studies suggest that FGFR-1 ectodomain shedding could play an important physiological role in both growth and development. 

## 4. Experimental Procedures

### 4.1. Plasmids and Reagents

The 155 amino-acid form of human recombinant FGF-2 was expressed in *Escherichia coli* [59] and purified using heparin-Sepharose affinity chromatography and ion-exchange chromatography, as previously described [60]. The FGFR-1/pcDNA3 mammalian expression plasmid was constructed by subcloning the human three Ig-domain FGFR-1(IIIc) gene [61] into the HindIII- BamH1 site of pcDNA3 (Invitrogen, San Diego, CA, USA). The mammalian expression plasmid—pSV2/dhfr—which expresses the dihydrofolate reductase gene, was a gift from Dr. Shunichi Shimasaki (University of California; San Diego, CA, USA). Horseradish peroxidase-conjugated goat anti-mouse IgG was purchased from Biorad (Hercules, CA, USA). Monoclonal antibodies (Mab6) were raised to the extracellular domain of the three Ig-like domain isoform of recombinant FGFR-1 produced in insect cells [48] and were purified from the conditioned media of hybridoma cells using protein G-Sepharose columns. FGFR-1 antibodies (anti-Flg, C-terminus) and ERK 1 (K-23) antibodies were purchased from Santa Cruz Biotechnologies (Santa Cruz, CA, USA). Antibodies to phospho-p44/42 MAP kinase (Thr202/Tyr204) were purchased from New England BioLabs (Beverly, MA, USA). Marimastat and CGS 27023A were generous gifts from Dr. Motowo Nakajima (Novartis; Takarazuka, Japan). 12-O-tetradecanoyl phorbol 13-acetate (TPA) was purchased from Sigma (St. Louis, MO, USA) and wheat germ lectin Sepharose was purchased from Amersham Pharmacia Biotech (Piscataway, NJ, USA). G_0_6983 was purchased from Calbiochem (La Jolla, CA, USA). SU5402 was a gift from Dr. Hideo Kimura (National Institute of Neuroscience, Tokyo, Japan).

### 4.2. Cell Culture and Transfections

All cell culture media was obtained from Life Technologies (Rockville, MD, USA), unless otherwise indicated. COS 7 cells were cultured in Dulbecco’s modified Eagle’s medium (high glucose) supplemented with 10% fetal calf serum (HyClone, Logan, UT, USA), and 100 units/mL penicillin G sodium and 100 µg/mL streptomycin sulfate. COS 7 cells were transfected in 100 mm plates by the high efficiency calcium phosphate precipitation method, according to standard procedures [62], or by lipofectamine, using the manufacturer’s protocol, and split into 60 mm plates for the analysis of FGFR-1 ectodomain shedding. CHO/dhfr- cells were cultured in Iscoves Modified Dulbecco’s Media (IMDM) containing 10% fetal calf serum, supplemented with 0.1 mM hypoxanthine (Life Technologies; Gibco BRL), 0.01 mM thymidine (Sigma Chemical; St. Louis, MO, USA), 100 units/mL penicillin G sodium, and 100 µg/mL streptomycin sulfate. NIH (3T3 cells were cultured in DMEM (high glucose) supplemented with 10% calf serum, 2 mM glutamine, 100 U/mL penicillin G sodium, and 100 µg/mL streptomycin sulfate. Adult bovine aortic endothelial cells were cultured in DMEM (low glucose) supplemented with 10% fetal bovine serum, 10 mM HEPES (4-(2-hydroxyethyl)-1-piperazineethanesulfonic acid), 2 mM glutamine, 0.1 mM nonessential amino acids, 100 units/mL penicillin G sodium, and 100 µg/mL streptomycin sulfate. 

### 4.3. Production of Stably Transfected FGFR-1 Expressing CHO Cell Lines

Two stably transfected CHO cell lines overexpressing the secreted form of the FGFR-1 receptor and the full-length three Ig-like domain isoform of FGFR-1(IIIc) were produced, defined as FGFR-1/pcDNA3 (h1/p3). The cell lines were produced by cotransfecting CHO/dhfr- cells with 20 μg of secreted FGFR-1 or full length FGFR-1/pcDNA3 and 1 μg of pSV2/dhfr using Lipofectamine, according to the manufacturer’s recommendations (Life Technologies; Rockville, MD, USA). The transfected CHO cells were switched to IMDM containing 10% dialyzed fetal calf serum and 0.01 µM methotrexate (Sigma Chemical; St. Louis, MO, USA). Multiple clones were isolated, amplified with increasing concentrations of methotrexate, and screened to select colonies expressing high levels of the soluble FGFR-1 receptors. The level of protein expression was determined by immunoblot analysis. Clones were expanded, adapted to serum-free SFX^TM^-CHO media (Hyclone; Logan, UT, USA), and grown to concentrations of 1–10 × 10^6^ cells/mL in 1 L Celline CL1000 flasks (IBS Integra Biosciences; Ijamsville, MD, USA). The serum-free conditioned media containing the shed or secreted soluble FGF receptors was collected three times weekly.

### 4.4. Cell Lysis, Immunoprecipitation, and Gel Electrophoresis

Transfected COS 7 cells were rinsed with cold PBS and lysed in 1% NP-40 lysis buffer (20 mM Tris (pH7.5), 150 mM NaCl, 1 mM DTT, 1% (*v*/*v*) NP-40, 0.5 mM PMSF, 20 µg/mL leupeptin, and 1 µg/mL aprotinin). The lysates were clarified by centrifugation and the proteins were immunoprecipitated by incubating the cell lysates with anti-FGFR-1 antibodies (Santa Cruz, CA, USA) at 4 °C. After 2 h, protein G-Sepharose beads (25 µL bead volume) were added and the samples were incubated for an additional 15 h at 4 °C. The immunoprecipitated proteins were extensively washed in lysis buffer and eluted by boiling in SDS sample buffer. The proteins were resolved by SDS-PAGE on an 8% polyacrylamide gel (Invitrogen, San Diego, CA, USA).

### 4.5. Immunoblotting

Proteins separated by SDS-PAGE (sodium dodecyl (lauryl) sulfate-polyacrylamide gel electrophoresis) were electrotransferred to nitrocellulose membranes and blocked in blocking buffer (5% (*w*/*v*) nonfat dried milk, 50 mM Tris (pH 7.5), 150 mM NaCl, and 0.05% (*v*/*v*) Tween-20) for 2 h at room temperature on a rocker. The membranes were probed with Mab6 (2 µg/mL) for 15 h at 4 °C, as previously described [26]. The immunoreactive proteins were detected by enhanced chemiluminescence (Pierce; Rockford, IL, USA). All immunoblotting originals can be found at Supplementary Materials.

### 4.6. Analysis of the Cleavage and Release of Soluble FGFR-1

COS 7 cells transfected with FGFR-1/pcDNA3 were rinsed three times with phosphate buffered saline (PBS) and incubated in 2 mL of serum-free OptiMem medium (Life Technologies, Gibco BRL) for 2–24 h, depending on the experiment, at 7% CO_2_ and 37 °C. The conditioned medium was collected and clarified by centrifugation to remove cellular debris and 1.8 mL was incubated with WGA (wheat germ agglutinin)-Sepharose beads (25 µL bead volume) overnight at 4 °C on a rocker to precipitate the FGFR-1 ectodomain. Following the incubation period, the beads were collected, washed three times in PBS, and boiled in SDS sample buffer. The shed FGFR-1 ectodomain was detected by immunoblot analysis. A semi-quantitative analysis of the FGFR-1 receptor signals was carried out using the public domain NIH Image program (developed at the U.S. National Institutes of Health.

We studied the regulation of soluble FGFR-1 shedding in COS 7 cells which were transiently transfected with FGFR-1/pcDNA3, and in CHO cells which were stably transfected with FGFR-1/pcDNA3. The cells were incubated in 2 mL of serum-free OptiMem medium either overnight or for 2–4 h in the presence of various dilutions of the following reagents: Human recombinant FGF-2 alone; the MMP inhibitors marimastat and CGS 27023A (0–400 μM); TPA (0–100 ng/mL) in the presence or absence of the protein kinase C inhibitor G_0_6983 (1 μM); and FGF-2 in the presence or absence of the FGF receptor tyrosine kinase inhibitor SU5402 (20 μM) or the protein kinase C inhibitor G_0_6983 (1 μM). The conditioned media containing the shed FGFR-1 ectodomain was collected and analyzed as described above.

### 4.7. Purification of Recombinant Soluble FGFR-1 Ectodomains

The shed FGFR-1 ectodomain in the conditioned media of FGFR-1/pcDNA3 cells was purified to homogeneity over an FGF/HS (heparin sulfate) affinity column [28,63]. Briefly, 3 mg of human recombinant FGF-2 was loaded on a 5 mL heparin-Sepharose Hi-Trap column (Amersham Pharmacia Biotech, Piscataway, NJ, USA) in 50 mM Hepes (pH 7.5), 0.5 M NaCl, and 10 mM DTT (dithiothreitol) and washed with 50 mM Hepes (pH 7.5) and 0.5 M NaCl (loading buffer). The FGFR-1 conditioned media was adjusted to 0.5 M NaCl, 0.3 mM PMSF (phenylmethylsulfonyl fluoride), pH 7.5; loaded over the FGF-2/HS column; and washed with loading buffer until the absorbance returned to the baseline. The FGFR-1 ectodomain was eluted with a linear gradient of Buffer A (20 mM NaAcetate pH 5.0, 0.1 M NaCl, 0.3 mM PMSF), containing 21% Buffer B (A + 3 M NaCl, 0.3 mM PMSF). The eluate containing the FGFR-1 ectodomain was dialyzed against PBS and concentrated using a stirred Ultrafiltration Cell concentrator (Millipore Corp., Bedford, MA, USA). Coomassie blue staining was performed using the GelCode Blue Stain Reagent (Pierce; Rockford, IL, USA). A typical yield was 40 ug of the shed FGFR-1 ectodomain from 100 mL of h1/p3 conditioned media.

We also purified the shed and secreted FGFR-1 ectodomains with WGA-Sepharose and/or DEAE-Sepharose columns. For this procedure, conditioned media from FGFR-1/pcDNA3 or secreted FGFR-1/pcDNA3 cell lines was passed over a WGA-Sepharose column (Vector Labs; Burlingame, CA, USA); rinsed with 20 mM Hepes (pH 7.5); and eluted with 20 mM Hepes, 2 mM EDTA (ethylenediaminetetraacetic acid), and 10% glycerol containing 0.5 M N-acetylglucosamine. The sample was diluted 1:1 with Buffer A (10 mM NaPO_4_, pH 8.0), loaded on a DEAE-Sepharose column, and eluted with a linear gradient of Buffer A containing 0.5 M NaCl. The fractions containing the shed FGFR-1 and secreted ectodomains were analyzed by SDS-PAGE in combination with immunoblotting and dialyzed against PBS. 

### 4.8. Cell Proliferation Assays

ABAE cells were plated in 24 well plates at 3000 cells/well. The cells were treated in duplicate in the presence or absence of 0.125 ng/mL of recombinant FGF-2 and various concentrations of purified soluble FGF receptors on day 1 and again on day 3. After five days in culture, the cells were washed with phosphate-buffered saline, incubated with 0.5% trypsin/EDTA and counted with a Coulter counter (Beckman Coulter; Miami, FL, USA).

### 4.9. Gel Invasion Angiogenesis Assays 

Adult bovine aortic endothelial (ABAE) cells were seeded onto collagen gels prepared with 500 µL Vitrogen 100 (Collagen Corp., Fremont, CA, USA) in 24-well tissue culture plates at 2 × 10^5^ cells/well in DMEM containing FGF-2 (500 ng) and secreted FGFR-1 receptor at doses of 0, 30, 300, and 3000 ng/mL. After seven days, the invasive capillary-like structures were photographed within the collagen gel using phase contrast microscopy. Capillary tube formation was quantified as the total area of vessel growth in three separate photographic fields for each condition using ImageJ software.

### 4.10. ^125^I-FGF-2 Ligand Binding Assays

^125^I-FGF-2 ligand binding assays were performed as previously described [64]. Swiss 3T3 fibroblasts were cultured to confluence in 24-well dishes. The cells were chilled to 4 °C and 300 μL of ice-cold dilutions of ^125^I-FGF-2 (2 ng/mL) in HEPES-buffered DMEM, 0.2% gelatin, and the soluble FGFR-1 ectodomains were added to the cells in triplicate and incubated for 2 h at 4 °C. The cells were washed once with 0.5 mL of PBS, and twice with 0.5 mL of 20 mM HEPES, 2 M NaCl, pH 7.5, to remove FGF-2 bound to low affinity sites, and then solubilized with 1% Triton X-100 buffer (1% Triton X-100, 50 mM HEPES, pH 7.5, 50 mM NaCl, 5 mM EDTA, 1 mM Na_2_VO_3_), and this was counted as FGF-2 bound to high affinity sites.

### 4.11. Measurement of the Stimulation of DNA Synthesis

Swiss 3T3 cells were grown in 96-well dishes (4000 cells/well) for 2 days in DMEM containing 10% calf serum, at which time they were confluent. After washing with serum-free DMEM, they were incubated for a further 2 days in DMEM with 0.5% calf serum. The rate of DNA synthesis was measured 24 h after the addition of FGF-2 and soluble FGF ectodomains by the addition of 0.2 mCi/well of [methyl-^3^H]thymidine (6.7 Ci/mole, ICN), followed by incubation for 5 h. The cultures were then processed for scintillation counting, as previously described [49].

### 4.12. Construction of FGFR-1 Mutants

The 3.5 kb cDNA fragment encoding human FGFR-1 in the mammalian expression vector pcDNA3 (FGFR-1/pcDNA3) was used to generate the mutants employed in this study. A 138 bp HincII-KpnI fragment containing a portion of the third Ig-domain and the transmembrane region was subcloned into pBluescript for PCR mutagenesis. The first mutant—Bingo (B1)—was made by deleting a stretch of seven amino acids called PAVMTSP (ProAlaValMetThrSerPro) containing the putative Val-Met cleavage site [26,47], located just eight amino acids before the transmembrane domain. The mutant P2 was constructed by deleting three additional amino acids, for a total deletion of 10 amino acids (EERPAVMTSP), and substituting a proline-glycine pair (CCG-GGT) to introduce conformational instability in the region of the cleavage site. The third mutant—L4—contained a deletion of 14 amino acids (LEALEERPAVMTSP) preceding the transmembrane region. The loss of a NaeI restriction site served as a marker to identify the mutants. 

The primers used to generate the mutants (upstream and downstream sequences) were as follows: 5′ CTGGAAGAGAGGCTGTACCTGGAGATC 3′ and 5′ CAGCCTCTCTTCCAGGGCTTC 3′ for Bingo (B1); 5′ CGGACCCAGGGCTTCCAGAACGGT 3′ and 5′ GTTCTGGAAGCCCTGGGTCCGCTGTACCTGGAGATCATC 3′ for P2; and 5′ AACGGTCAACCATGCAGAGTG 3′ and 5′ GCATGGTTGACCGTTCTGTACCTGGAGATCATC 3′ for L4. The mutations were confirmed by sequencing before subcloning back into the HindIII-KpnI site of pcDNA3/FGFR-1 (h1/p3) for further studies.

## Data Availability

The data is contained within the article or supplementary material.

## Supplementary Materials

The following are available online at https://www.mdpi.com/1422-0067/22/5/2712/s1.

## Abbreviations

FGF	fibroblast growth factor
FGFR	fibroblast growth factor receptor
PKC	protein kinase C
MMP	matrix metalloprotease
TNF	tumor necrosis factor
CHO	Chinese hamster ovary
ADAM	a disintegrin and metalloprotease
MAP kinase	mitogen activated protein kinase
ERK	extracellular signal regulated kinase
EGF	epidermal growth factor
TIMP	tissue inhibitor of metalloprotease
TPA	12-O-tetradecanoyl phorbol 13-acetate
ABAE cells	adult bovine aortic endothelial cells
DMEM	Dulbecco’s modified Eagle media
IMDM	Iscoves modified Dulbecco’s media
SDS-PAGE	sodium dodecyl sulfate-polyacrylamide gel electrophoresis
WGA	wheat germ agglutinin
CSF	colony stimulatory factor
PCR	polymerase chain reaction
