# Supplementary figures and images for: Constitutive and Regulated Shedding of Soluble FGF Receptors Releases Biologically Active Inhibitors of FGF-2
Source: Int J Mol Sci. 2021 Mar 8;22(5):2712. doi: 10.3390/ijms22052712 (PMC7962449; doi:10.3390/ijms22052712)

064-141

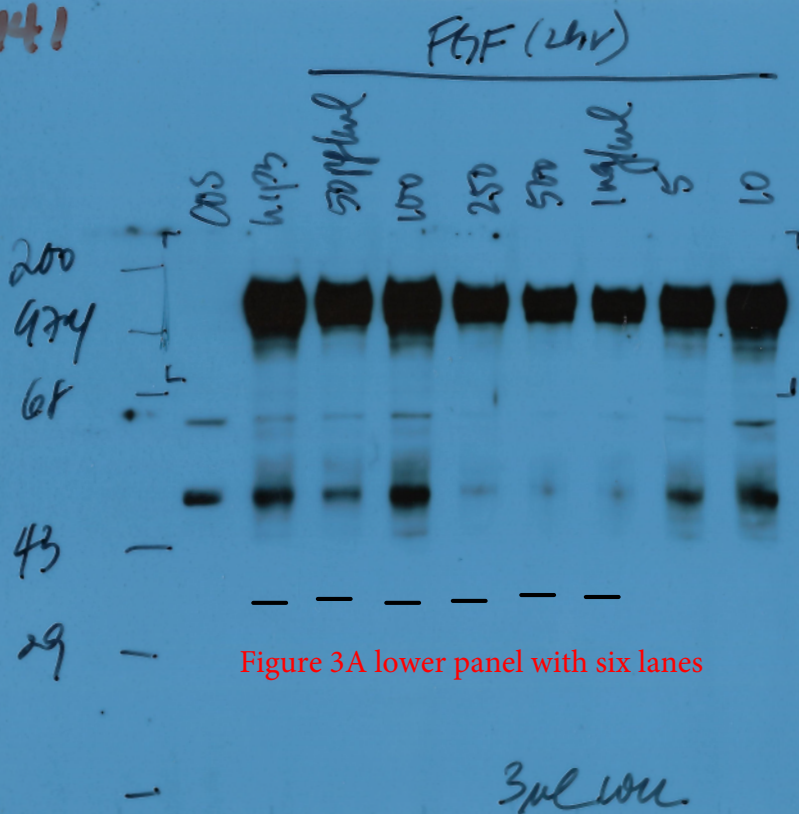

Supplement: Supplementary file 1 [file ijms-22-02712-s001.zip › Figure 3A_lower.pdf]

Figure 9B. All six lanes here

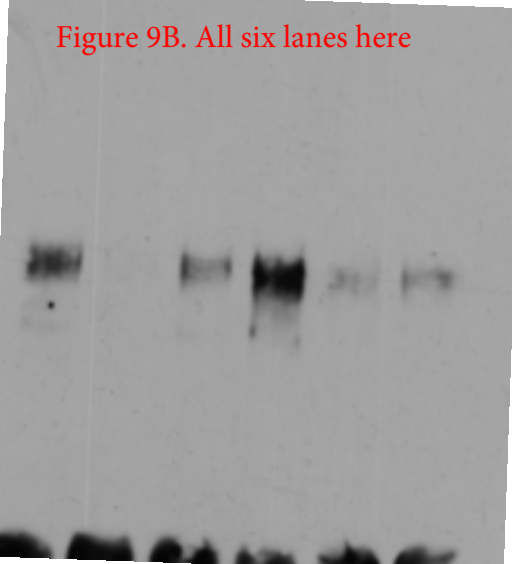

Supplement: Supplementary file 1 [file ijms-22-02712-s001.zip › Figure 9B.pdf]

Figure 9A. Five lanes here.

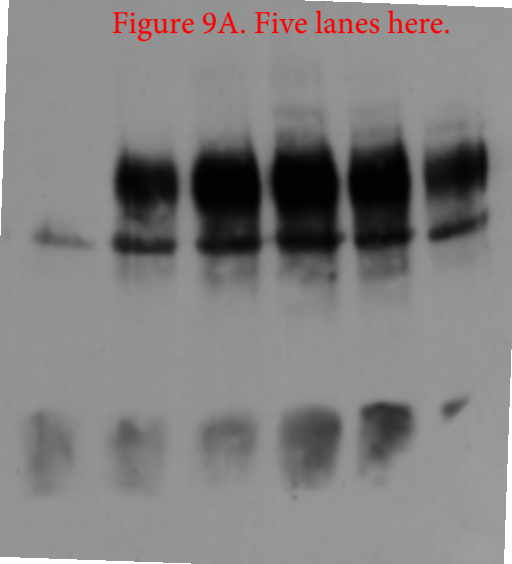

Supplement: Supplementary file 1 [file ijms-22-02712-s001.zip › Figure 9A.pdf]

053-237

Slide 3  
Maximastab

Figure 7- All Lanes of both gels

1/25

Slide 2  
CGS

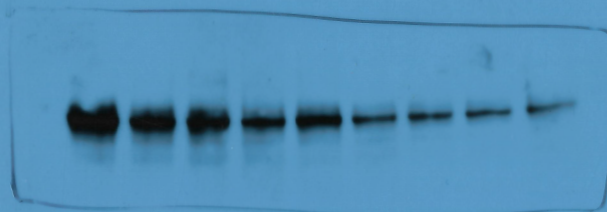

chl. 1 10 50 100

[M]  
μ

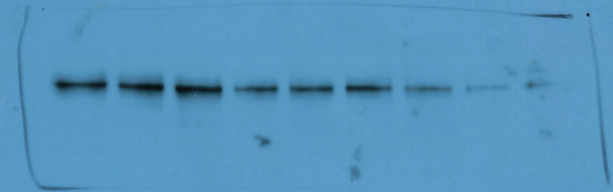

chl. 4 40 200 400

[M]  
μ

Supplement: Supplementary file 1 [file ijms-22-02712-s001.zip › Figure 7.pdf]

053-281

2.8 - cm

Figure 3B, upper panel with 7 lanes

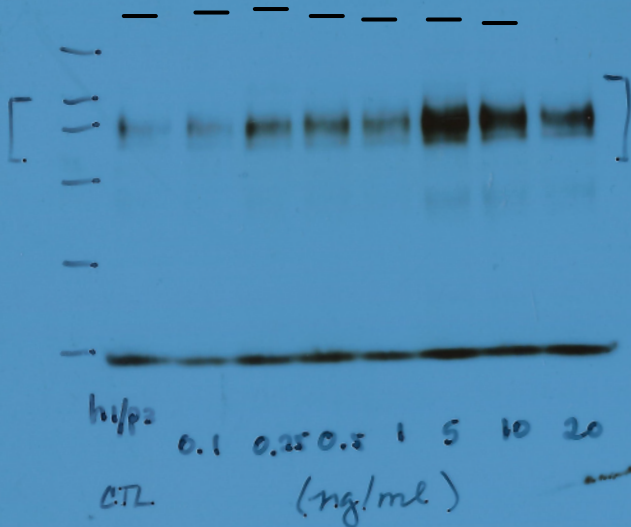

Supplement: Supplementary file 1 [file ijms-22-02712-s001.zip › Figure 3B_upper_band.pdf]

053-291

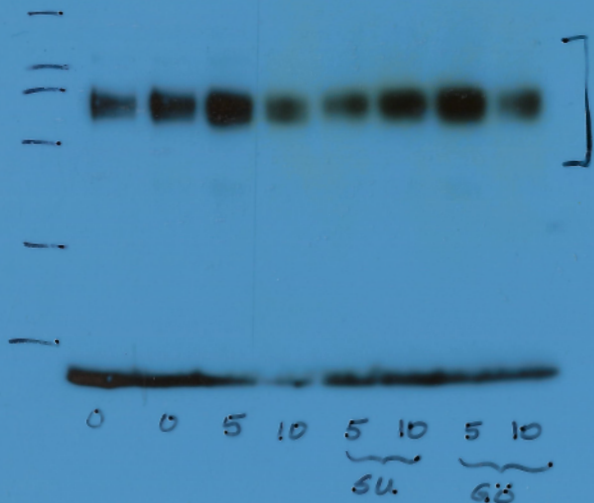

Figure 10A. Four lanes shown with arrows

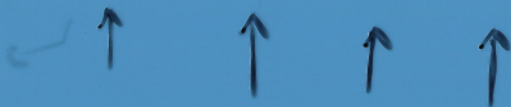

Supplement: Supplementary file 1 [file ijms-22-02712-s001.zip › Figure 10A.pdf]

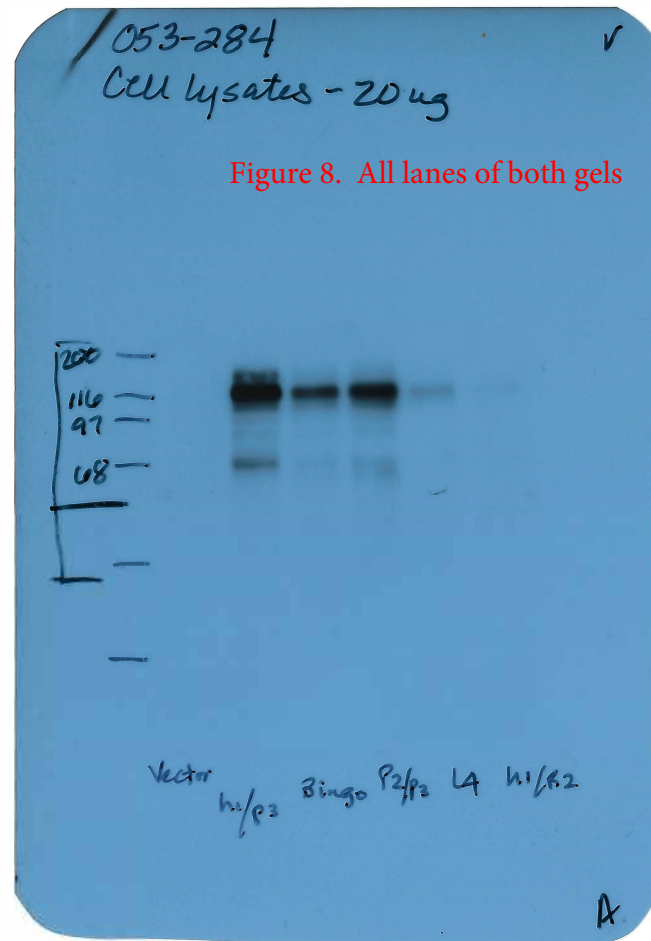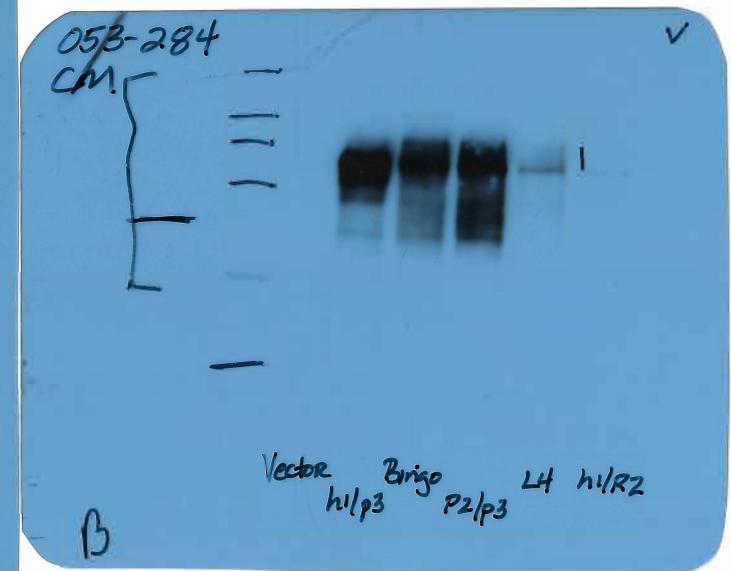

Figure 8

Supplement: Supplementary file 1 [file ijms-22-02712-s001.zip › Figure 8.pdf]

FEF (500pfu/ml)

constitutive cleavage

3/15/77

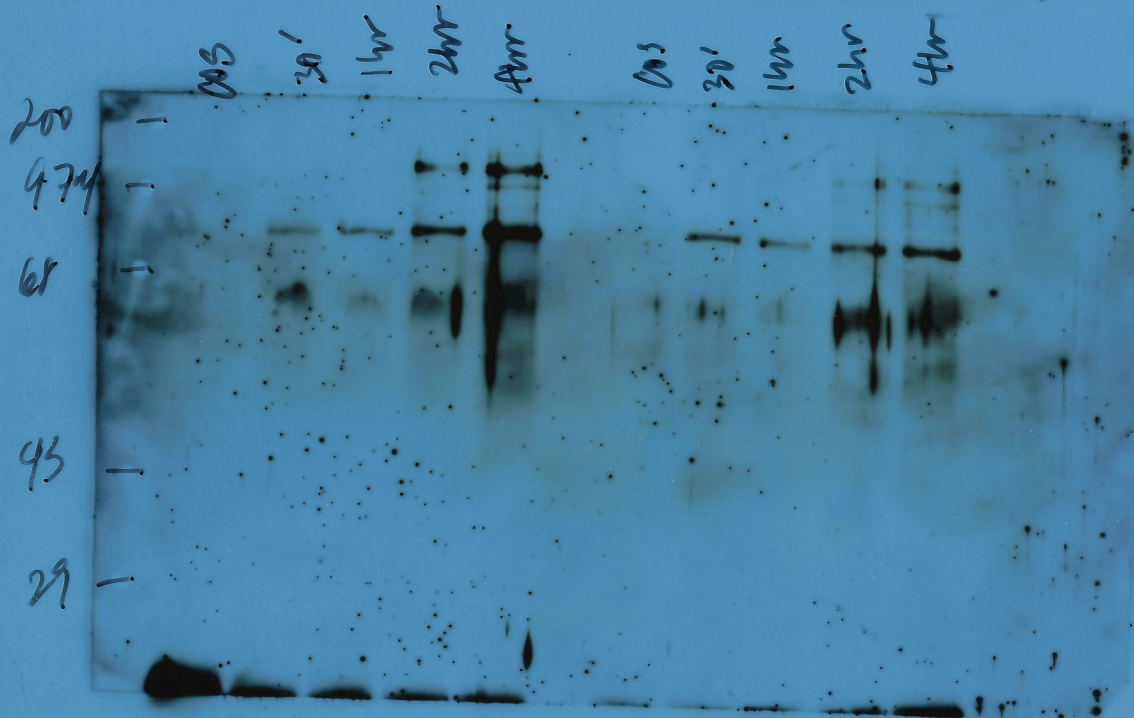

FEF time course  
trachinot  
hip3 or  
water on

LAB 6 TH

Supplement: Supplementary file 1 [file ijms-22-02712-s001.zip › Figure 2_constitutive_shedding.pdf]
